# Tracing Metabolic Fate of Mitochondrial Glycine Cleavage System Derived Formate In Vitro and In Vivo

**DOI:** 10.3390/ijms21228808

**Published:** 2020-11-20

**Authors:** Yee-Ling Tan, Nga-Lai Sou, Feng-Yao Tang, Hsin-An Ko, Wei-Ting Yeh, Jian-Hau Peng, En-Pei Isabel Chiang

**Affiliations:** 1 Food Science and Biotechnology, National Chung Hsing University (NCHU), Taichung 402, Taiwan; tyling38@gmail.com (Y.-L.T.); looksusan2013@gmail.com (N.-L.S.); khsinan@gmail.com (H.-A.K.); chetbaker04567321@gmail.com (W.-T.Y.); jianhau.peng@gmail.com (J.-H.P.); 2 Innovation and Development Center of Sustainable Agriculture (IDCSA), National Chung Hsing University (NCHU), Taichung 402, Taiwan; 3 Department of Nutrition, China Medical University, Taichung 402, Taiwan; vincenttang@mail.cmu.edu.tw; 4 Microbial Genomics Ph.D. Graduate Program, National Chung Hsing University (NCHU), Taichung 402, Taiwan; 5 Department of Food Science and Biotechnology, National Chung Hsing University, Taichung 402, Taiwan

**Keywords:** one-carbon metabolism, serine, glycine, glycine cleavage system, stable isotopic tracers, metabolic kinetics

## Abstract

Folate-mediated one-carbon (1C) metabolism is a major target of many therapies in human diseases. Studies have focused on the metabolism of serine 3-carbon as it serves as a major source for 1C units. The serine 3-carbon enters the mitochondria transferred by folate cofactors and eventually converted to formate and serves as a major building block for cytosolic 1C metabolism. Abnormal glycine metabolism has been reported in many human pathological conditions. The mitochondrial glycine cleavage system (GCS) catalyzes glycine degradation to CO_2_ and ammonium, while tetrahydrofolate (THF) is converted into 5,10-methylene-THF. GCS accounts for a substantial proportion of whole-body glycine flux in humans, yet the particular metabolic route of glycine 2-carbon recycled from GCS during mitochondria glycine decarboxylation in hepatic or bone marrow 1C metabolism is not fully investigated, due to the limited accessibility of human tissues. Labeled glycine at 2-carbon was given to humans and primary cells in previous studies for investigating its incorporations into purines, its interconversion with serine, or the CO_2_ production in the mitochondria. Less is known on the metabolic fate of the glycine 2-carbon recycled from the GCS; hence, a model system tracing its metabolic fate would help in this regard. We took the direct approach of isotopic labeling to further explore the in vitro and in vivo metabolic fate of the 2-carbon from [2-^13^C]glycine and [2-^13^C]serine. As the 2-carbon of glycine and serine is decarboxylated and catabolized via the GCS, the original 13C-labeled 2-carbon is transferred to THF and yield methyleneTHF in the mitochondria. In human hepatoma cell-lines, 2-carbon from glycine was found to be incorporated into deoxythymidine (dTMP, dT + 1), M + 3 species of purines (deoxyadenine, dA and deoxyguanine, dG), and methionine (Met + 1). In healthy mice, incorporation of GCS-derived formate from glycine 2-carbon was found in serine (Ser + 2 via cytosolic serine hydroxy methyl transferase), methionine, dTMP, and methylcytosine (mC + 1) in bone marrow DNA. In these experiments, labeled glycine 2-carbon directly incorporates into Ser + 1, A + 2, and G + 2 (at C2 and C8 of purine) in the cytosol. It is noteworthy that since the serine 3-carbon is unlabeled in these experiments, the isotopic enrichments in dT + 1, Ser + 2, dA + 3, dG + 3, and Met + 1 solely come from the 2-carbon of glycine/serine recycled from GCS, re-enters the cytosolic 1C metabolism as formate, and then being used for cytosolic syntheses of serine, dTMP, purine (M + 3) and methionine. Taken together, we established model systems and successfully traced the metabolic fate of mitochondrial GCS-derived formate from glycine 2-carbon in vitro and in vivo. Nutritional supply significantly alters formate generation from GCS. More GCS-derived formate was used in hepatic serine and methionine syntheses, whereas more GCS-derived formate was used in dTMP synthesis in the bone marrow, indicating that the utilization and partitioning of GCS-derived 1C unit are tissue-specific. These approaches enable better understanding concerning the utilization of 1C moiety generated from mitochondrial GCS that can help to further elucidate the role of GCS in human disease development and progression in future applications. More studies on GCS using these approaches are underway.

## 1. Introduction

Abnormal folate metabolism is associated with numerous human diseases, such as cancer [1], and folate-mediated one-carbon (1C) metabolism is a major target of many therapies in human diseases [2]. Abnormal glycine metabolism has been reported in human pathological conditions, including obesity, diabetes, neurological disorders, and others [3]. Glycine participates in numerous metabolic pathways, including the syntheses of serine, sarcosine, purines, creatine, heme group, glutathione, and collagen [3]. Humans can synthesize glycine from glyoxylate, from glucose via serine, betaine, and threonine, as well as during L-carnitine synthesis. Mitochondrially derived formate is critical in mammalian metabolism and development [4]. Oxidation of serine 3-carbon is a major pathway for 1C unit generation through mitochondria [5,6]. Cytoplasmic 1C metabolism uses mitochondrially derived formate for nucleotide and methionine biosynthesis. The interconversion of serine and glycine makes glycine a secondary source for 1C units [7]. In humans, the glycine cleavage system (GCS) accounts for a substantial proportion of whole-body glycine flux [8], implying that GCS could serve as an important route for the generation of 1C units.

**Figure 1:**
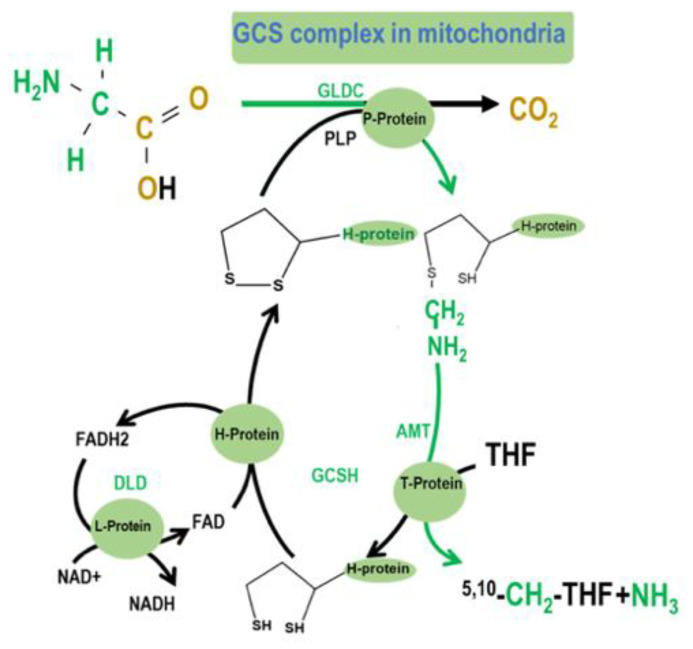
The glycine cleavage system (GCS) is a multienzyme complex that mediates the breakdown of glycine in mitochondria. Glycine decarboxylase (*GLDC*; glycine dehydrogenase) catalyzes the first step of glycine cleavage, releasing CO_2_, with an accessory H protein GCSH, to which the amino methyl moiety is transferred [10]. The subsequent reaction is catalyzed by amino methyltransferase (AMT) that transfers the second 1C unit to tetrahydrofolate (THF), generating 5,10-CH_2_-THF. The further steps of mitochondrial folate 1C metabolism ultimately supply 1C units as formate to the cytoplasm for nucleotide biosynthesis and methylation reactions [11].

GCS is a multienzyme complex that generates folate activated 1C units by catalyzing the reversible oxidation of glycine to carbon dioxide, ammonia, and 5,10-methylene tetrahydrofolate (5,10-CH_2_-THF) [9]. GCS comprises four proteins: The P-protein (*GLDC*), which catalyzes the pyridoxal-phosphate–dependent decarboxylation of glycine; the H-protein (GCSH), a lipoic acid-dependent hydrogen carrier; the T-protein (AMT), which exhibits THF-dependent aminomethyltransferase (AMT) activity; and the L-protein (DLD), a lipoamide dehydrogenase (Figure 1). The complex localizes to the inner-mitochondrial membrane. GCS catalyzes glycine degradation to CO_2_ and ammonium, while tetrahydrofolate (THF) is converted into 5,10-CH_2_-THF. When glycine is decarboxylated and catabolized via GCS in the mitochondria, the 2-carbon of glycine can be transferred to tetrahydrofolate (THF) to yield 5,10-CH_2_-THF and subsequently formate, which can be used as a building block for cytosolic folate-mediated 1C metabolism. 

The glycine cleavage rate observed in humans implies a high rate of 5,10-CH_2_-THF formation from glycine; hence, GCS may account for a substantial proportion of whole body glycine flux [8]. Deletion of the *AMT* gene in the GCS results in neural tube defects in the fetus that can be partially ameliorated by maternal methionine supplementation that is not sufficient to rescue the viability. Deficient in GCS activity, due to later *GLDC* deletion in live mice, also caused neural tube defects that can be rescued by maternal formate supplementation [12]. *GLDC* is critical for promoting 1C unit production and preventing methylglyoxal accumulation in pluripotent cells [13]. These studies suggest GCS activity is critical in formate homeostasis. 

*GLDC* has been proposed to be a metabolic oncogene that links glycine metabolism with tumorigenesis [14,15]. On the other hand, in certain cancers, 1C units’ supply for purine synthesis is absolutely dependent on serine rather than glycine uptake. In cancer cells, increasing glycine progressively inhibits cell growth [16]; it was proposed that the acquisition of enhanced *GLDC* activity may provide an advantage during tumor initiation, possibly degrading excess glycine that might otherwise accumulate and cause growth inhibitory effects [16]. In these cases, excess glycine is not used to generate 1C units for purine synthesis, but is converted to serine that depletes rather than replenishes 1C pools [16]. Regardless, GCS could serve as a major route for the generation of 1C units in humans, the contribution of the recycled glycine 2-carbon from glycine decarboxylation in cellular 1C metabolism is not fully elucidated. Its significance in human disease development and progression needs to be studied further. 

Regulation of folate-mediated cellular 1C metabolism reactions could be specific to tissue/cell type and the stage of transformation [17,18]. The liver is the major site of methionine and glycine utilization, whereas serine is consumed by many peripheral organs [19]. The metabolic fate of glycine 2-carbon in the liver, and the contribution of GCS in hepatic methyl group supply and homeostasis is unknown. Distinct expression pattern of tissue-specific 1C metabolic enzymes may impact the activation of GCS, as well as the directionality of carbon flow in different cell compartments [17]. We postulated that the metabolic fate of 2-carbon from glycine generated by hepatic GCS differs significantly from that in highly proliferating tissues, such as bone marrow. Due to limited tissue availability in humans, tissue-specific data from in vivo labeling experiments and the requirement for mitochondrial GCS-derived 1C unit in tissues have not been fully elucidated. Characterization of the metabolic fate of glycine 2-carbon in different tissues/cell-types can certainly help in this regard. Developing a model system to trace the metabolic fate of glycine 2-carbon in different tissues and cell-models can better elucidate the essentiality of GCS in humans.

**Figure 2:**
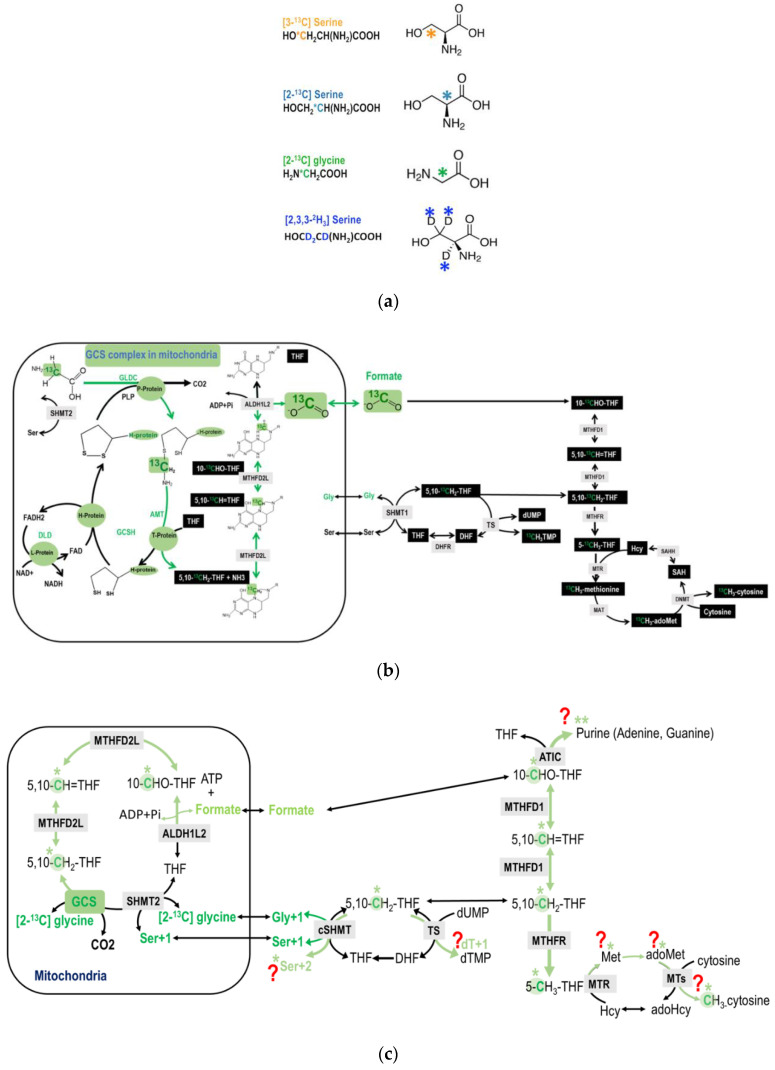
(**a**) The chemical structures of the tracers. (**b**) An overview of the potential metabolic fate of glycine 2-carbon during glycine decarboxylation. The glycine 2-carbon is exported from the mitochondria as formate. (**c**) An illustration of the detailed potential metabolic route of the GCS recycled glycine 2-carbon that enters cytosolic folate-mediated 1C metabolism. The original glycine tracer is marked in green, and the recycled glycine 2-carbon from the GCS is marked in light green with a * mark. The ** marked in light green indicates that, theoretically the GCS recycled carbon can be incorporated into the purine ring at 2 possible positions (C2 and C8). This is just to show the potential metabolic fate. the majority M + 2 enrichments in purines (dA + 2 and dG + 2) come from the direct incorporation of glycine carbon-2 at position 4 and 5.

Labeled glycine at 2-carbon was given to humans to investigate its direct incorporations into purines and excreted as uric acid [20,21] rather than investigating its metabolic route via the mitochondria GCS. Previous studies using isotopically labeled glycine investigated the incorporations of glycine in purine [20,21], serine glycine partitioning [22,23], and the CO_2_ production [8] during mitochondria glycine decarboxylation. Moreover, attention has been focused on the β-carbon (3-carbon) of serine as its hydroxymethyl group is released from the mitochondria as formate and serves as the main 1C supply in the cytosol [2,24,25,26,27,28]; and relatively less attention was drawn on the metabolic route of serine 2-carbon. Theoretically, the 2-carbon of serine can also generate formate by its conversion to glycine. We proposed direct approaches to trace the metabolic fate of glycine 2-carbon and serine 2-carbon from GCS and explored the contribution of GCS-derived 1C units for nucleotide and amino acid biosyntheses in vitro and in vivo. As the 2-carbon of glycine and serine is decarboxylated and catabolized via the GCS, the original ^13^C-labeled glycine 2-carbon is transferred to THF and yields 5,10-CH_2_-THF, 1C units can be further used cytosolic 1C metabolism (Figure 2a,b).

## 2. Results

The chemical structures of the tracers used in the present study are shown in Figure 2a. Formate is generated from the 2-carbon of glycine via mitochondrial GCS; and cytoplasmic 1C metabolism uses mitochondrially derived formate for cytosolic 1C metabolism (Figure 2b).

### 2.1. Tracing Metabolic Fate of C2-Carbon of Glycine via GCS in 1C Metabolism 

In the labeling experiments, isotopic enrichments from the tracers are calculated as molar ratios of labeled to non-labeled isotopomers after correction for the natural abundance of stable isotopes. The M + 1 is the isomer of the metabolite that contains 1 extra mass unit, the M + 2 is the isomer of the metabolite that contains 2 extra mass units, etc. It is noteworthy that in this case, isotopic enrichments in deoxythymidine (dTMP) M + 1 and in serine M + 2 only appear when the 2-carbon of glycine/serine goes through GCS, re-enters the cytosolic 1C metabolism as mitochondria-derived formate, and is used for serine and dTMP synthesis in the cytosol. In other words, dTMP M + 1 and in serine M + 2 solely comes from 2-carbon of glycine/serine via GCS activity (Figure 2c). 

The GCS-derived 1C moiety exported from the mitochondria as formate might further be used in cytosolic folate-mediated 1C metabolism. In the labeling experiment using [2-^13^C]glycine, as the glycine is decarboxylated and catabolized via the GCS in the mitochondria, the original 13C-labeled glycine 2-carbon is transferred to tetrahydrofolate (THF) to yield 13C-labeled methyleneTHF, that will ultimately produce 13C-labeled formate. Theoretically, this 1C unit can be utilized in cytosolic biosyntheses for nucleotides, serine, methionine, S-adenosylmethionine (adoMet), and deoxymethyl-cytosine (mC) (Figure 2c).

**Figure 3:**
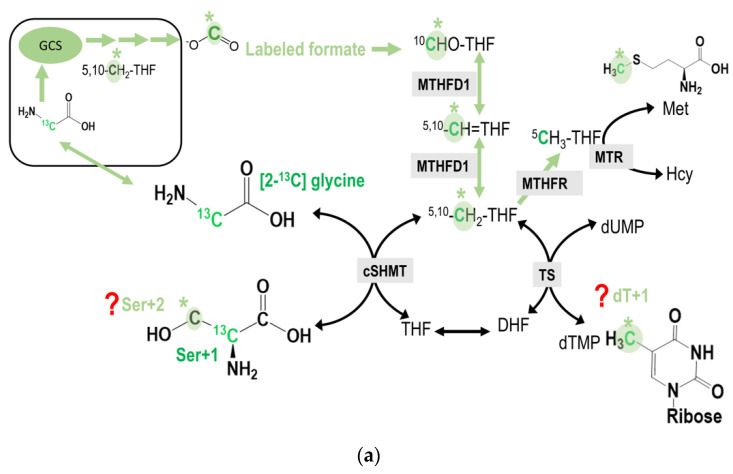

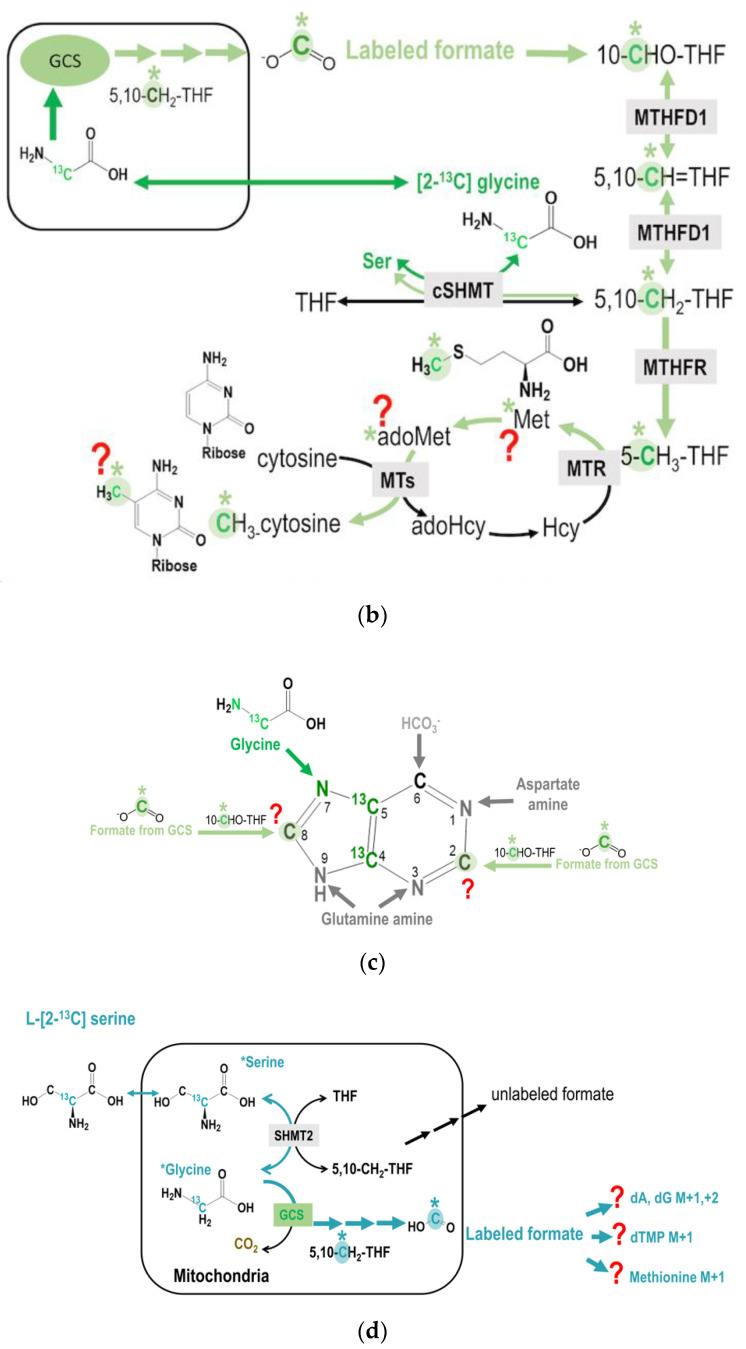
(**a**) the potential incorporation of C2-carbon of glycine into cytosolic serine and dTMP syntheses. The original glycine tracer is marked in green, and the recycled glycine 2-carbon from the GCS is marked in light green with a * mark. The M + 2 species of serine (Ser + 2) can only be detected when the GCS-derived formate from the C2-carbon of [2-^13^C]glycine is further used for the cytosolic serine synthesis from the [2-^13^C]glycine via cytosolic serine hydroxymethyl-transferase (cSHMT). On the other hand, the M + 1 species of deoxy-thymidine (dTMP, dT + 1) can only be detected when the GCS-derived formate from the C2-carbon of [2-^13^C]glycine is subsequently transferred to dUMP via methyleneTHF in the cytosol. (**b**) The potential incorporation of C2-carbon of glycine into transmethylation. The original glycine tracer is marked in green, and the recycled glycine 2-carbon from the GCS is marked in light green with a * mark. The GCS-derived C2-carbon from [2-^13^C]glycine may ultimately be incorporated into the methyl group of deoxy-cytidine in the DNA. (**c**) The potential incorporation of C2-carbon of glycine into the purine ring at C2 and/or C8. The original glycine tracer is marked in green, and the recycled glycine 2-carbon from the GCS is marked in light green with a * mark. (**d**) In the L-[2-^13^C] serine labeling experiments, any enrichment detected in M + 1 species of methionine and thymidine would solely reflect the mitochondrial 1C unit coming from the C2-carbon of serine (via mSHMT) and/or glycine (via GCS activity). The original serine tracer is marked in turquoise, and the recycled serine 2-carbon from the GCS is marked with a *.

The M + 2 species of serine (Ser + 2) can only be detected when the GCS-derived formate from the C2-carbon of [2-^13^C]glycine is further used for the cytosolic serine synthesis from the [2-^13^C]glycine via cytosolic serine hydroxymethyl-transferase (cSHMT). The predicted carbon flow for this reaction is shown (Figure 3a). The M + 1 species of deoxy-thymidine (dTMP, dT + 1) can only be detected when the GCS-derived formate from the C2-carbon of [2-^13^C]glycine is subsequently transferred to dUMP via methyleneTHF in the cytosol (Figure 3a).

The M + 1 species of methionine (Met + 1) can only be detected when the GCS-derived formate from the C2-carbon of [2-^13^C]glycine is used for generating 5-methyl-THF(5-CH_3_THF) in the cytosol that is subsequently used for methionine synthesis via methionine synthase (MTR). If the methyl group is used for adoMet synthesis, it can be incorporated into the methyl group of deoxy-cytodine (mC) in the DNA (Figure 3b).

Since 2-carbon of glycine is originally designated for the 4-carbon and 5-carbon in the purine ring in *de novo* purine synthesis, if the GCS-derived formate is taken and incorporated into the purine ring at positions 2 and 8, enrichments in M + 3 or even M + 4 of deoxyadenine (dA) and deoxyguanine, (dG) might be detected. These enrichments can only occur when the GCS-derived formate is incorporated into the purine ring with already labeled carbon 4 and 5 from [2-^13^C]glycine (Figure 3c).

### 2.2. Tracing Metabolic Fate of Serine 2-Carbon via GCS in 1C Metabolism

In the labeling experiment using L-[2-^13^C] serine tracer, the 2-carbon of serine can be transferred to glycine in both the cytosol and mitochondria. However, the only glycine within the mitochondria can enter the GCS that produces 1C units and is exported as formate into the cytosol. As a result, any enrichment detected in M + 1 species of methionine and thymidine, would solely reflect the mitochondrial 1C unit coming from the C2-carbon of serine (via mSHMT) and then glycine (via GCS) activity (Figure 3d).

### 2.3. Metabolic Fate of 2-Carbon from Glycine and Serine via GCS in Cell Models

A total of 6 hepatocyte-derived cell-lines were examined in the present study (Table 1). Among them, three cell-lines, L02, An042, and GNMT+ express GNMT; the other cell-lines, HepG2, Skep-1, and GNMT− do not express GNMT. Not all cell-lines examined were found to utilize the 2-carbon of glycine via GCS under these experimental conditions, regardless of GNMT expression (Table 1). Enrichments in dT + 1 were detected in L02, An042, GNMT+, and GNMT− cell-lines when incubated with [2-^13^C]glycine. In a separate experiment using L-[2-^13^C]serine, enrichments in dT + 1 were also detected in An042, GNMT+, and GNMT− cell-lines, consistent with the fact that these cell-lines have active GCS. In both experiments, more enrichments were found in dT + 1 compared to that in Ser + 2 in all cell-lines with GCS (Table 1). When comparing the enrichments in dT + 1 and Ser + 2 from the same tracer, there is a significant difference in the ratio between Ser + 2 and dT + 1: GNMT− is ~79%; GNMT+ is ~71%; An042 is ~50% (calculated based on data from Table 1). These results may imply the directionality of cSHMT among different cell-lines. 

### 2.4. Restoring GNMT Expression Decreased Formate Generation from Glycine 2-Carbon via GCS 

Glycine-N methyltransferase (GNMT) is a major hepatic enzyme that converts *S*-adenosylmethionine (adoMet) to *S*-adenosylhomocysteine (adoHcy), while generating sarcosine from glycine. We postulated that GCS is activated in cell-lines with defective GNMT function or when excessive glycine is supplied. GNMT expressing cells (GNMT+) and GNMT-null cells (GNMT−) were cultured in minimum essential medium (MEM) with B_12_, or in MEM with B_12_ and non-essential amino acid (NEAA), as described in the experiment procedures under the Method section. Cells were then supplemented with [2-^13^C]glycine or L-[2-^13^C]serine for 72 h before harvest. Our data demonstrated that GNMT expression may impact folate-mediated 1C metabolism via GCS. Nutrition supply also affected the utilization of 2-carbon of glycine (Table 2). Specifically, GCS activity was significantly reduced when NEAA was supplied into the media (Table 2). 

**Figure 4:**
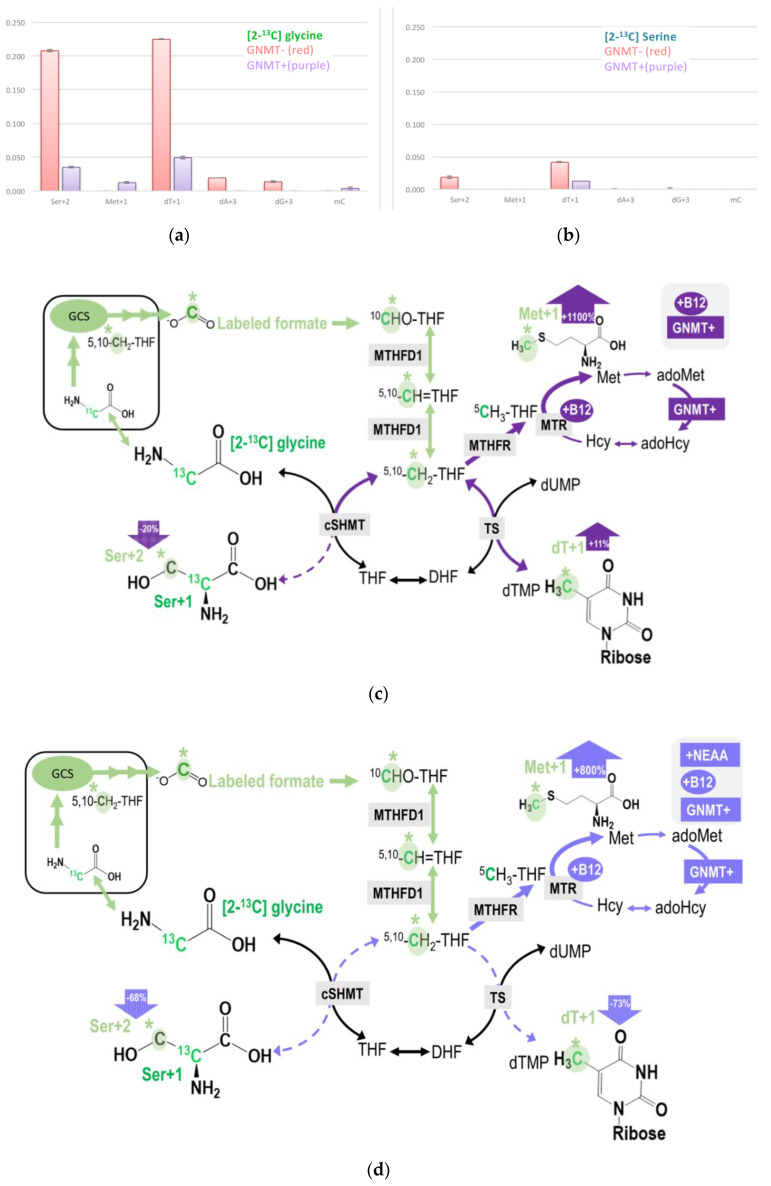
(**a**,**b**) Restoring GNMT expression significantly decreased isotopic enrichments in target metabolites originated from the C-2 carbon of glycine via GCS. (**c**) Vitamin B12 supplementation increased GCS-derived formate utilization in dTMP and methionine syntheses, but decreased this 1C used in cytosolic serine formation from glycine in GNMT+ cells. (**d**) NEAA supply decreases GCS-derived formate utilization in both GMNT+ and GNMT− cells. The original glycine tracer is marked in green, and the recycled glycine 2-carbon from the GCS is marked in light green with a * mark for (**c**,**d**).

Isotopic enrichments derived from glycine 2-carbon via mitochondrial GCS are compared between GNMT+ and GNMT− cells (Figure 4a, Table 2). Theoretically, glycine 2-carbon can be detected as the M + 2 specie of serine (Ser + 2), M + 1 species of methionine (Met + 1), methyl-cytosine (mC + 1), dTMP (dT + 1), and M + 3 species of purines (dA + 3, dG + 3). GNMT− cells had 3-8-fold more enrichments in dTMP + 1 and 3-5-fold more in enrichments Ser + 2 compared to GNMT+ cells. Isotopic enrichments in the M + 3 species of purines (dA + 3 and dG + 3) were only detected in transformed human hepatoma cell-line without GNMT expression (GNMT−), but not in the GNMT+ cells. These data imply that GNMT− cells utilized much more glycine 2-carbon compared to GNMT+ cells.

In contrast, isotopic enrichment in the methionine was only detected in GNMT+, but not GNMT− cells (Figure 4a, Table 2). Compared to [2-^13^C]glycine labeling experiment (Figure 4a), much less enrichment was detected in the target metabolites in the labeling experiment using L-[2-^13^C]serine tracer (Figure 4b). These observations may provide insights into the serine and glycine conversion in the mitochondria.

Data from both labeling experiments demonstrated that GNMT cells utilized more 2-carbon from glycine via GCS in nucleotide and cytosolic serine generation, presumably due to more intracellular glycine accumulation. Restoring GNMT expression (GNMT+) significantly decreased enrichments detected in the target metabolites, indicating that formate generation from glycine 2-carbon via GCS is a lot less when GNMT function is intact (Figure 4a,b). Despite much more enrichment being found in dT + 1 and Ser + 2 in GNMT− cells, the GCS-derived 1C was not detected in Met + 1 or mC + 1 in this cell-line. These results are not surprising because folate-dependent remethylation from the C-3 carbon of serine is almost undetectable in the GNMT− cell-line [26,27]. Data from this experiment indicate the significant role of GNMT on the utilization of mitochondrial GCS-derived 1C units. 

With respect to nutritional supply, vitamin B12 supplementation increased GCS-derived formate utilization in dTMP and methionine syntheses, but decreased this 1C used in cytosolic serine formation from glycine in GNMT+ cells (Table 2, Figure 4c). Furthermore, the NEAA supply decreases GCS-derived formate utilization in both GMNT+ and GNMT− cells (Table 2, Figure 4d).

### 2.5. Tracing Metabolic Fate of 3-Carbon of Serine in 1C Metabolism 

Folate-mediated serine oxidation is believed to be a major pathway for the flux of 1C units through mitochondria [5]. The 3-carbon of serine serves as the major 1C source in the folate cycle that can subsequently be used for methionine synthesis via folate-dependent homocysteine remethylation, and for purine and dTMP syntheses. SHMT catalyzes the transfer of the 3-carbon of serine to THF to form glycine and 5,10-CH_2_THF. This reversible reaction occurs in the mitochondria and in the cytosol. The metabolic fate of serine 3-carbon was assessed by labeling experiments using [3-^13^C]serine as the tracer [24]. We also used [2,3,3-^2^H_3_]serine to trace the relative contribution of the cytosolic and mitochondrial 1C metabolism. The cytosolic route is catalyzed by cytosolic SHMT that generates [2H_2_]-5,10-CH_2_-THF (M + 2). The mitochondrial route requires a dehydrogenase step where one deuterium (2H) is released, resulting in [2H_1_]-5,10-CH_2_-THF (M + 1). The relative abundance of M + 1 and M + 2 in dTMP and methionine reflect the partitioning between mitochondria and cytosolic metabolic fluxes [30,31].

### 2.6. Utilization of the Glycine 2-Carbon in dTMP Synthesis via Mitochondrial GCS In Vivo 

**Figure 5:**
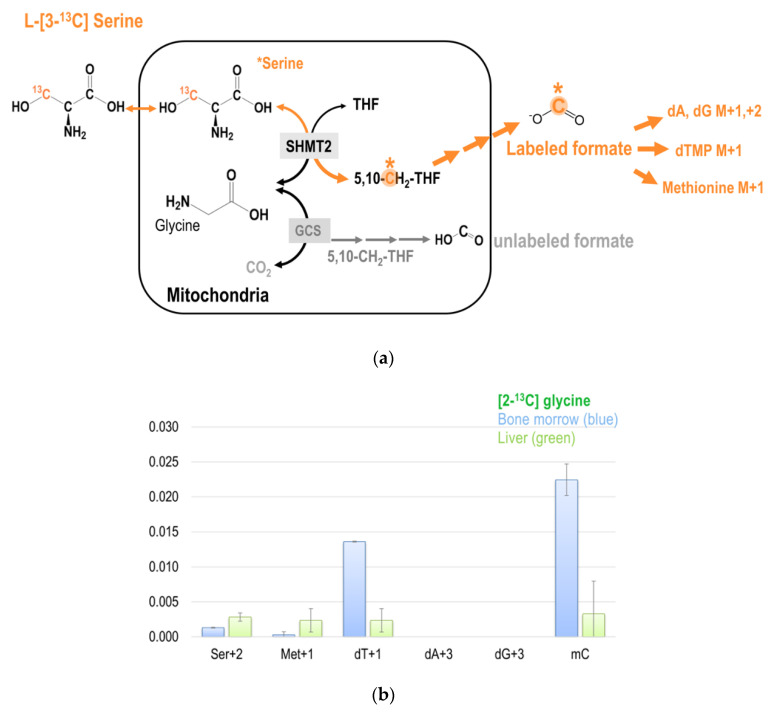

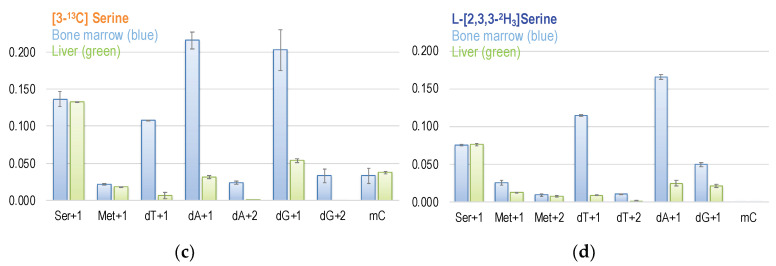
(**a**) The metabolic fate of serine 3-carbon was assessed by labeling experiments using [3-^13^C]serine as the tracer. The 3-carbon of serine serves as the major 1C source in the folate cycle that can subsequently be used for methionine synthesis via folate-dependent homocysteine remethylation, and for purine and dTMP syntheses. (**b**) Enrichments in target metabolite serine, methionine, dTMP, and deoxy-methylcytosine (mC) in the bone marrow and the liver from mice received [2-^13^C]glycine tracer for six days. These approaches enable a better understanding of the utilization of 1C moiety generated directly from mitochondrial GCS in vivo. (**c**,**d**) Enrichments in target metabolite serine, methionine, dTMP, purines, and deoxy-methylcytosine (mC) in the bone marrow and the liver from mice received [3-^13^C]serine (**c**) and [2,3,3-^2^H_3_]-serine (**d**). The 3-carbon of serine is marked in orange with a * mark.

The M + 2 species of serine (Ser + 2) can be detected only when the glycine 2-carbon is converted to formate by GCS in the mitochondria that were later incorporated into a pre-labeled serine from [2-^13^C]glycine by cSHMT. Unlike the transformed human hepatoma cell-line GNMT− that glycine 2-carbon can be incorporated into purines (dA + 3 and dG + 3) in the DNA (Figure 4a,b), no enrichments were found in M + 3 species of purines in healthy mice tissues (Figure 5b). Interestingly, more enrichments from glycine 2-carbon were found in Ser + 2 and Met + 1, but much less enrichment was found in dT + 1 in the liver, indicating that the partitioning of GCS-derived 1C unit utilization is tissue-specific (Figure 5b). 

## 3. Discussion

### 3.1. Metabolic Fate of C2-Carbon of Glycine and Serine via GCS 

The first study using [2-^13^C]glycine to quantify human GCS indicated that GCS may account for over a third of whole body glycine flux [8]. Yet, the metabolic fate of glycine 2-carbon in 1C metabolism has not been fully elucidated in specific tissues. Previous studies focused on the CO_2_ production route from glycine 1-carbon [8], purine synthesis [20,21] in humans, or cellular serine glycine interconversion in vitro [22] without further determination of the ultimate fate of recycled glycine 2-carbon from GCS in the tissues. Theoretically, the recycled glycine 2-carbon during glycine decarboxylation can re-enter 1C metabolism by forming 5,10-methyleneTHF in the mitochondria that is finally exported from the mitochondria as formate. The contribution of glycine 2-carbon in GCS-derived formate dependent pathways have not been fully investigated, particularly in well-differentiated hepatic tissues and highly proliferating bone marrow. Our present study demonstrates that GCS-derived formate from glycine 2-carbon can be incorporated into cytosolic serine, methionine, and deoxymethylcytosine (MC) in a tissue-specific manner. We postulated and proved that the GCS-derived formate from the glycine 2-carbon contributes to the 1C mediated transmethylation and methylation of DNA. GCS-derived formate is incorporated in the mC of bone marrow DNA. Our experimental approaches also proved that the GCS can serve as an important source of glycine-derived formate and used in the dTMP synthesis that was predicted by mathematical modeling [32].

*De novo* dTMP synthesis requires 5,10-methyleneTHF that can be generated from THF by two pathways. One is from formate, ATP, NADPH, and THF that is catalyzed by methylenetetrahydrofolate dehydrogenase 1 (MTHFD1). The other is by transferring the hydroxymethyl group of serine to THF that is catalyzed by SHMT [31] (Figure 2c). The mitochondrial formate production is a key process for the endogenous generation of folate-related 1C moieties [6], and that is utilized to meet the cytosolic 1C demands in the cytosol, including nucleotide [11] and methionine synthesis. Isotopic serine labeling experiments indicated that approximately 90% of the used 1C units in dTMP comes from mitochondria formate [29,31]. Our experimental approaches discovered that the GCS-derived formate is used more in dTMP compared to that used in cytosolic serine synthesis in the bone marrow.

In addition to cytosolic nucleotide and methionine synthesis, mitochondria released formate can also be used to re-synthesize serine via cytosolic 1C metabolism [33]. Cells may use the reversible SHMT to synthesize serine in one compartment and catabolize it in another. In the primary culture of fetal ovine hepatocytes, approximately 30% of serine biosynthesis is derived from glycine, primarily via the combined action of GCS and SHMT [34]. The contribution of GCS-derived formate on cytosolic serine generation is also unknown. Numerous studies indicated that the net flux through cytosolic SHMT (cSHMT) mainly occurs in the glycine to serine direction [5,35,36]. In the present study, more enrichment was detected in dT + 1 compared to that in Ser + 2 in all cell-lines using [2-^13^C]glycine or L-[2-^13^C]serine tracers. Enrichments in dTMP + 1 were detected in L02, An042, GNMT+, and GNMT− cell-lines when incubated with the above tracers. In contrast, enrichments in Ser + 2 from L-[2-^13^C]serine were only observed in the GNMT− cell-line (Figure 4a,b). L02, An042, GNMT+, and GNMT− effectively used GCS-derived formate for cytosolic dTMP, but not for serine synthesis in the cytosol. Results from these experiments reflect the directionality of cSHMT and utilization of GCS-derived formate under such study conditions. The 1C moiety is preferentially used for generating CH_2_THF that is further used for dTMP synthesis. Figure 3a and Figure 4c,d illustrate the impacts GNMT expression, NEAA, and vitamin B12 supply on the partitioning of mitochondrial GCS-derived formate (either entering dTMP or cytosolic serine synthesis from glycine). 

### 3.2. Restoring GNMT Expression Decreased Formate Generation from Glycine 2-Carbon via GCS 

In healthy humans receiving primed, constant infusion of [1,2-^13^C_2_] glycine, GCS accounted for more than one-third of whole-body glycine flux [37]. We aimed to use cell and mouse models to further investigate the metabolic fate of glycine 2-carbon in dTMP, purine, and methionine syntheses. It is noteworthy that isotopic enrichments in dT + 1, Ser + 2, A + 3, and G + 3 only appear when the 2-carbon of glycine/serine goes through GCS, re-enters the cytosolic 1C metabolism as mitochondria-derived formate, and is used for serine, dTMP, and purine synthesis. 

Most transformed HCC cell-lines are defective with GNMT; we postulated that glycine accumulation, due to defected GNMT, may activate glycine catabolism by GCS. However, we discovered that the GCS activity, estimated by the utilization of glycine 2-carbon, varies among different liver-derived cell-lines: GCS activity was not detected in Skep1 or HepG2 under our culture conditions. In contrast, low enrichments from [2-^13^C]glycine and L-[2-^13^C]serine were detected in dTMP in normal adult human hepatocyte L02, suggesting the presence of low GCS activity. On the other hand, substantial GCS activity were found in 3 HCC derived cell-lines: An042 (with GNMT and MAT1a transfection); GNMT+ (with GNMT transfection); GNMT− (with vector-only, as the null cell-line for GNMT+). Among these three cell-lines, cells with both MAT1a and GNMT (An042) appeared to have lower GCS, followed by cell-line expressing GNMT (GNMT+); GNMT− cells had the most GCS activity detected, indicated by the enrichments in dT + 1 and Ser + 2, dA + 3, and dG + 3 (Table 1). These results imply that restoring GNMT and MAT1a may decrease formate generation from 2-carbon of glycine via GCS, but future studies are needed to confirm the role of these genes on GCS activity. The variations among these cancer and non-cancer cell-lines indicate that more studies are warranted to explore the role of GCS activity and its association with cancer cell metabolism. Despite that much more enrichment was found in dT + 1 and Ser + 2 in GNMT− cells, the GCS-derived 1C was not detected in Met + 1 or mC + 1 in this cell-line. We previously reported that GNMT expression is critical for the methyl group homeostasis in these cell models; GNMT− cells had minimal or undetectable remethylation fluxes via MTR [26,27]. Expression of GNMT in human hepatoma cells without GNMT helped maintain the methyltransferase activity and possibly maintain DNA methylation [26]. No enrichments are detected in methionine in GNMT− cells, due to the impaired homocysteine remethylation in this cell-line. 

### 3.3. Nutritional Supply Affect the Utilization of GCS-Derived Formate 

GCS activity is closely related to glycine supply. The total free glycine concentration in the human liver is ~2.5 mmol/L [38]. Glycine is compartmentalized, with a concentration of 0.83 mmol/L in the cytosol and 1.86 mmol/L in the mitochondria [32]. The K_m_ of glycine decarboxylase (*GLDC*) is higher (6 mmol/L) [39] than that of the tissue glycine concentrations. Mitochondrial glycine decarboxylation and the concurrent 5,10-methyleneTHF generation by the GCS is sensitive to mitochondrial glycine concentration because of the relatively higher K_m_ compared to the mitochondrial glycine concentration. 

The contribution of GCS-derived formate under various nutritional supply on dTMP synthesis has not been fully investigated previously. Vitamin B12 may affect formate metabolism and utilization. During cobalamin deficiency in rats, the disposition of 10-formyl-tetrahydrofolate carbon is shifted in favor of formate production. It was suggested that this could be a mechanism that generates more 1C units for the replenishment of the adoMet pool, which is depleted in this condition [40]. Our study demonstrates that nutritional supply strongly affects the utilization of a 2-carbon of glycine by GCS. In particular, vitamin B12 supplementation significantly increased the utilization of GCS-derived formate for dTMP synthesis (dT + 1) in both GNMT+ and GNMT− cells, but not in cytosolic glycine conversion to serine (Ser + 2) in these cells. Compared to the same cells cultured in MEM, vitamin B12 supplementation increased dT + 1 enrichment from 2-carbon by 11% in GNMT+ and by 17% in GNMT− cells. It was reported that the nuclear 5-methylTHF trap resulting from vitamin B12 depletion suppresses *de novo* dTMP biosynthesis [41]. Our findings support the critical role of vitamin B12 in dTMP synthesis and further suggest that GCS-derived formate could contribute significantly to dTMP synthesis in some cells. On the other hand, vitamin B12 supplementation decreased cytosolic glycine conversion to serine (Ser + 2) by 20% in GNMT+, but had little impact on Ser + 2 in GNMT− cells. Such a difference may result from the activated remethylation in GNMT+ cells. We previously reported that GNNT expression regulates homocysteine remethylation and methyl group homeostasis in HepG2 cells [26]. GNMT expression and B12 supplementation re-direct the utilization of GCS-derived formate among 1C metabolic pathways. The promotion of homocysteine remethylation by vitamin B12 in GNMT+ cells reduces the use of GCS-derived formate in cytosolic glycine conversion to serine, but increases that in dTMP synthesis. As more 1C is used in transmethylation, fewer 1C moieties are used in cytosolic serine synthesis from glycine as more 1C are used in dTMP synthesis (Figure 4c). On the other hand, the NEAA supply suppressed GCS activity, and such suppression is stronger in cells with GNMT expression (Table 2C). When MEM media was supplemented with vitamin B12, alanine, asparagine, aspartic acid, glutamate, and proline, utilization of glycine 2-carbon derived formate via GCS decreased drastically, especially in GNMT+ cells (Figure 4d). Enrichments in Ser + 2 and dTMP decreased by 68% and 73% in GNMT+, whereas enrichments in Ser + 2 and dTMP decreased by 47% and 28% in GNMT− cells. Compared to the GNMT−cells, expression of GNMT decreased Ser + 2 enrichments by 62–77% and decreased dTMP enrichments by 67–88% under various conditions (Table 2). 

The contribution of GCS and the metabolic fate of 2-carbon from glycine in the liver is unclear, as sampling from human tissue is too invasive. Considering the liver has a unique metabolic role, we proposed that the metabolic fate of 2-carbon from glycine differs from that of other tissues, especially from the highly proliferating tissues, such as the bone marrow. Compared to bone marrow, we discovered slightly more enrichment in the M + 1 and M + 2 species of serine synthesized from [2-^13^C]glycine in the liver (Figure 5b). In contrast, more enrichment in the M + 1 in dTMP was detected in the bone marrow (Figure 5b). These interesting findings suggest distinct carbon flows among these two tissues, and support our hypothesis that the metabolic fate of 2-carbon from glycine differs between liver and bone marrow. Low expressions of MTHFD2 and MTHFD1L in rodent liver [42,43] may also account for the minimal enrichments found in the M + 1 specie of dTMP in the liver; no M + 3 species of purines were detected either. With very low mitochondrial MTHFD activity in the liver, mitochondrial 1C units may not be fully oxidized and exported as formate, but rather be used to synthesize serine from glycine in the mitochondria. The synthesized serine can then be exported to the cytosol, where the 1C unit generated by cSHMT can be used for transmethylation reactions. Our unpublished data on mouse models indicate at least some cytosolic 1C production from serine. On the other hand, the formate generated from GCS is used for cytosolic serine synthesis from 2-carbon, generating Ser + 2. 

### 3.4. The Partitioning of GCS-Derived 1C Unit Utilization is Tissue-Specific 

The [2,3,3-^2^H_3_]serine labeling experiments indicate that approximately ~90% of the formate comes from mitochondria; the contribution of serine 3-carbon in mitochondrial formate is much more than that of glycine 2-carbon. The enrichment in dT + 1 from [3-^13^C]serine is 7.7-fold that from [2-^13^C]glycine in the bone marrow and ~3-fold in the liver (Figure 5b,c). We calculated and compared the mitochondria-derived formate from serine 3-carbon and from glycine 2-carbon via GCS; the enrichment in dTMP + 1 from is 6.9-fold that in the bone marrow and 2.7-fold that in the liver. These observations are comparable to the report from Brosnan et al., stating that the rate of formate production from serine is six-fold the formate production from glycine [6]. Although all tissues can produce and export formate from the mitochondria that is further used to synthesize nucleotides and methionine, the partitioning of 1C units differs among tissues. Earlier studies on the nutrient requirements of activated human lymphocytes demonstrated that serine is the main donor of 1C units in proliferating normal cells [44,45,46]. In non-proliferative adult tissues, such as the liver, 1C metabolism mainly supports the synthesis and homeostasis for the pools of adoMet-bound 1C units; it also maintains the homeostasis of nucleotides and amino acids, and controls the utilization of dietary choline, serine, and glycine as substrates for ATP and NADPH generation [47].

Interestingly, more enrichment from glycine 2-carbon was found in hepatic Ser + 2 and Met + 1 and less in the bone marrow, indicating that the partitioning of GCS-derived 1C unit utilization is tissue-specific (Figure 5b). Infusion of [2,3,3-^2^H]serine in human subjects produces serum methionine and liver-synthesized apoprotein B100 in both the M + 1 and M + 2 species, indicating at least some cytosolic 1C production from serine [48]. We suggest that methyleneTHF is preferentially used for dTMP synthesis in highly proliferative tissues like bone marrow. On the other hand, in non-proliferating tissues like the liver, the 1C unit from methyleneTHF might be utilized to synthesize serine and/or methionine. The metabolic switch of methyleneTHF has been reported in Raji cells exposed to excessive methionine [49]. When more methyleneTHF is used in serine synthesis that is needed for homocysteine transsulfuration, fewer 1C units are available for methyleneTHF-dependent dTMP synthesis. Such directionality may account, in part, for the different utilization of GCS-derived 1C unit between liver and bone marrow. 

### 3.5. Limitations of the Study

There are limitations to the current study. First, unlike the infusion protocol that provides a strictly constant rate and a fixed amount of tracer to each animal, the tracer consumption by diet cannot be 100% controlled during the labeling period. Animals on the infusion labeling protocol are sacrificed at various time points up to the enrichments achieve the plateau from the tracer, and the plateau enrichment of stable isotope-labeled amino acids and their metabolic products and the corresponding flux values can be calculated as μmol·h^−1^·kg^−1^. Without infusion, we cannot calculate the corresponding rate; yet, we can still effectively trace the ultimate metabolic fate of glycine carbon-2. Moreover, to overcome the potential confounding effects resulted from various tracer consumptions, the labeling diet provided (4 g per mouse per 24 h) was divided into small parts that were given every 3–4 h during the labeling periods to reduce the variation in tracer consumption. 

Second, non-essential amino acid precursors generated from regular metabolism may also supply for the intracellular amino acid pool for protein and DNA synthesis during the labeling period. For instance, serine tracer can be diluted by unlabeled serine generated from glycolysis (3-phosphoserine, hydroxy-pyruvate) [22]. Glycine is generated from serine via both mitochondria and cytosolic SHMT, and also is produced from glyoxalate. The endogenous produced amino acid(s) may compete with the exogenous amino acid tracer(s), and therefore, “dilute” the labeled precursors and result in lower labeled-carbon incorporation in the target compound. Furthermore, the endogenously produced (unlabeled) metabolites from different pathways may dilute the enrichments detected in the target compound. Such dilution can make it challenging in detecting the enrichments from the recycled 2-carbon generated from GCS, particularly in non-proliferating tissues, such as the liver. The enrichments of dT + 1 was much lower in the liver compared to that in the bone marrow. 

Nevertheless, our approach is still effective and useful to investigate the overall incorporation of glycine in the protein and DNA. Our approach also enables one to investigate how gene expression and nutrient supply alter the utilization of [2-^13^C]glycine and [3-^13^C]serine in *de novo* protein and DNA synthesis during the labeling period. In conclusion, we provide a powerful approach to discover the ultimate metabolic fate of carbon 2 of glycine in vivo.

## 4. Materials and Methods

### 4.1. Chemicals and Materials

Percholoric acid (J.T. Backer, Center Valley, PA, USA), Chloroform (J.T. Backer, Center Valley, PA, USA), Isopropanol (Merk, Whitehouse Station, NJ, USA), Methanol (ECOH, Taizhong, Taiwan), L-homocysteine (Sigma, St. Louis, MO, USA), L-cysteine (Sigma, St. Louis, MO, USA), 1-Heptanesulfonic acid sodium salt (Sigma, St. Louis, MO, USA) SDS (Merck, Whitehouse Station, NJ, USA), Tris Base (Merck, Whitehouse Station, NJ, USA), hydrochloric acid (Sigma, St. Louis, MO, USA), Nuclease P1 (Wako Co, Osaka, Japan). [2-^13^C]glycine, L-[2-^13^C] serine, L-[3-^13^C] serine, [2,3,3-^2^H_3_]-serine, L-[5,5,5-^2^H_3_]leucine, and were purchased from Cambridge Isotopes Laboratories (Cambridge Isotope Laboratories, Woburn, MA, USA). The chemical structures of the tracers used are shown in Figure 2a.

### 4.2. Cell-Lines and Culture Conditions

A total of 6 hepatocyte-derived cell-lines were examined in the present study. HepG2 cells (Bioresource Collection and Research Center, Taiwan), SK-HEP-1 (ATCC HTB-52), was obtained from American Type Culture Collection (ATCC) (Manassas, VA, USA). Adult human hepatic cell-line L02 was kindly provided by Dr. Shuang-En Chuang at the National Institute of Cancer Research, Miaoli County, Taiwan). L02 expresses MAT1A, betaine-homocysteine methyltransferase (BHMT), and methionine synthase (MTR), but not MAT2A [50,51], which is expected to better reflect normal hepatocyte metabolism than cancer cell-lines [52]. To elucidate how GNMT and MAT1a affect glycine 2-carbon metabolism, stable cell-lines were established from the human hepatoma cell-line HepG2 that has diminished GNMT, but retains morphological and biochemical characteristics of normal human hepatocytes [26,53]. One stable cell-line was established by cotransfecting pGNMT and pTK-Hyg plasmid (Clontech, Palo Alto, CA, USA) DNAs that represented a model with normal GNMT function (GNMT+). The negative control cell-line, GNMT− was established by stable transfection with pFLAG-CMV-5 [53]. The establishment of stable cell-lines was described in detail previously, and GNMT expression was confirmed by western blot analyses [53]. The full-length cDNA clone of human *MAT1A* was cloned into the mammalian expression vector pcDNA3. *MAT1A* expression plasmid or empty control vector pcDNA3 was delivered into a stable HepG2 clone that expresses GNMT by Lipofectamine 2000 and G418 selection (unpublished work). Cells were grown in MEM media containing 10% FBS (Gibco, Gaithersburg, MD, USA), penicillin (100,000 units/L), streptomycin (100 mg/L), amphotericin (0.25 mg/L). Cells were incubated in 5% CO_2_ at 37 °C, and the media were replaced every 72 h.

### 4.3. Stable Isotopic Experiments In Vitro

To compare the utilization of carbon C2-carbon of serine and glycine among different cell-lines, cells were cultured in minimum essential medium (MEM), supplemented with vitamin B12 alone with non-essential amino acid (NEAA) and the designated serine or glycine tracers. GNMT overexpression cells (GNMT+) and GNMT null cells (GNMT−) were cultured in MEM, supplemented with or without vitamin B12 and NEAA containing 0.281 mM L-alanine, 0.379 mM L-asparagine, 0.510 mM L-Glutamic Acid, 0.348 mM L-Proline, and 0.226 mM L-aspartic acid. The nutrient concentrations used were comparable to those in the standard αMEM media. Cells were supplemented with [2-^13^C]glycine (0.667 mM, 100% of total glycine) or L-[2-^13^C]serine (0.667 mM, 100% of total serine), and L-[5,5,5-^2^H_3_] leucine (190 μM).

In the labeling experiments, isotopic enrichments from the tracers are calculated as molar ratios of labeled to non-labeled isotopomers after correction for the natural abundance of stable isotopes. The M + 1 is the isomer of the metabolite that contains 1 extra mass unit, the M + 2 is the isomer of the metabolite that contains two extra mass units. L-[5,5,5-^2^H_3_] leucine was included in determining any changes in protein turnover [2,26]. The metabolic fate of 2-carbon of glycine was investigated by [2-^13^C]glycine. The GCS-derived 1C moiety is exported from the mitochondria as formate. As glycine is decarboxylated and catabolized via the GCS in the mitochondria, the original ^13^C-labeled glycine 2-carbon is transferred to THF to yield ^13^C-labeled methyleneTHF, which ultimately produces ^13^C-labeled formate. Theoretically, this 1C unit can be utilized in cytosolic biosyntheses for nucleotides, serine, methionine, adoMet, and mC. The metabolic fate of 2-carbon of serine was investigated by L-[2-^13^C]serine. The serine to glycine conversion occurs in both cytosol and mitochondria, and the 2-carbon of serine is transferred to glycine by SHMT. The only glycine within the mitochondria enters GCS that produces 1C units and is exported as formate into the cytosol. As a result, the enrichments detected in the M + 1 specie of methionine and dTMP would solely reflect the mitochondrial 1C unit coming from the 2-carbon of serine and then glycine (via GCS) activity. The predicted potential utilization of glycine 2-carbon (Figure 2c and Figure 3a–c) and serine 2-carbon (Figure 3d) are discussed in detail in the Results section above.

### 4.4. Tracing Metabolic Fate of Glycine 2-Carbon, Serine 2-Carbon, and 3-Carbon In Vivo

The animal protocol was approved by the Institutional Animal Care and Use Committee of National Chung Hsing University. Sixteen four-week-old female C57BL/6J mice were obtained from the National Laboratory Animal Center, NLAC (Taipei, Taiwan). Mice had access to sterilized water ad libitum and were provided an AIN-93M diet (Dyets, Bethlehem, PA, USA). Food intake and body weight was measured individually every 2–3 days throughout the study period. All mice were raised under a specific pathogen-free conditions in 20–25 °C. The lighting was operated on a 12-h light–dark cycle. In vivo studies were performed to trace the metabolic fate of glycine 2-carbon, serine 3-carbon used for DNA and protein synthesis in animals before the termination of the study. During this period, mice were fed a modified L-amino acid defined diet with the same amino acid composition as the AIN-93M diet, except for the substitution of designated tracer: [2-^13^C]glycine (for 144 h), L-[3-^13^C] serine (for 72 h) or L-[2,3,3-^2^H_3_]serine (for 72 h). The labeling diets were divided into small parts that were provided every 4 h during the labeling periods, to ensure the tracers were slowly and evenly consumed during the labeling period. Mice were sacrificed by isoflurane at the end of the labeling period after overnight fasting for 12 h. Liver tissues were immediately excited upon sacrifice. Bone marrow cells were collected, washed by PBS. All tissues were stored in −80 °C until analysis [52]. The predicted potential utilization of serine 3-carbon (Figure 5a) is discussed in the Results section above. 

### 4.5. Gas Chromatography/Mass Spectrometry Analysis

Cytosolic free amino acids from cells or tissue samples were extracted using 0.4 M ice-cold perchloric acid (PCA) [18]. Cellular protein were hydrolyzed in 6N HCl under vacuum. The amino acids were purified by cation-exchange chromatography [54]. Amino acids were converted to heptafluorobutyryl-propyl ester derivatives [28] and separated on an HP-5MS column (30 m × 0.25 nm). Isotopic enrichment was determined in electron capture negative ionization mode by gas chromatography-mass spectrometry (GC-MS) using a model 6890 GC and model 5975C MS (Agilent, Palo Alto, CA, USA), as described previously [24,25,31].

Genomic DNA was isolated and purified, as described previously [24,25]. The DNA samples were dried and hydrolyzed in formic acid under vacuum then derivatized by N, O- Bis- [trimethylsilyl] trifluoroacetamide 1% trimethyl-chlorosilane and acetonitrile. Isotopic enrichments of ^13^C_1_-serine in deoxyadenylate (dAMP, dA) and deoxyguanylate (dGMP, dG), and deoxythymidine (dTMP, dT), and deoxymethyl-cytosine (mC) were determined in positive ionization mode by GC/MS, as described previously [24,25,31].

### 4.6. Statistical Analysis

Comparisons of the means between the control and the treatment groups were determined using the Student’s t-test. Results are expressed as mean ± SD. All statistical analyses were performed with SYSTAT 11.0 Windows ^TM^ (Systat software Inc., Richmond, CA, USA). For all analyses, the results were considered statistically significant if *p* values were <0.05. A trend of difference with a *p* value < 0.1 is also reported.

## 5. Conclusions

Our study provides new information about glycine 2-carbon and GCS. We established model systems and successfully traced the metabolic fate of the mitochondrial glycine cleavage system derived formate in vitro and in vivo. GNMT expression and nutritional supply significantly alter formate generation from glycine 2-carbon via GCS. More GCS-derived formate was used in hepatic serine and methionine synthesis compared to that in the bone marrow, indicating that the partitioning of GCS-derived 1C unit utilization is tissue-specific. These approaches enable better understanding concerning the utilization of 1C moiety generated from mitochondrial GCS, and can help to further elucidate the role of GCS in human disease development and progression in future applications. More studies on GCS using these approaches are underway. 

## Figures and Tables

**Table 1:** Incorporations of carbon-2 from [2-^13^C]glycine and L-[2-^13^C]serine in dTMP, serine, and methionine ^1^^,^^2^.

				From GCS Derived Formate ^3^		
[2-^13^C]-glycine	**Leu + 1 + 2 + 3**	**Gly + 1**	**Ser + 1 ^4^**	**Ser + 2** ^**4**^	**dT + 1** ^**4**^	**Met + 1** ^**4**^
L02	0.262 ± 0.001	0.573 ± 0.018	0.250 ± 0.002	0.000 ± 0.000	0.003 ± 0.001	0.000 ± 0.000
Skep1	0.211 ± 0.004	0.641 ± 0.014	0.116 ± 0.001	0.000 ± 0.000	0.000 ± 0.000	0.002 ± 0.002
HepG_2_	0.216 ± 0.001	0.573 ± 0.005	0.306 ± 0.003	0.000 ± 0.000	0.001 ± 0.001	0.000 ± 0.000
An042	0.213 ± 0.002	0.436 ± 0.010	0.296 ± 0.006	**0.008 ± 0.001**	**0.016 ± 0.003**	0.000 ± 0.000
GNMT+	0.239 ± 0.006	0.489 ± 0.010	0.377 ± 0.004	**0.035 ± 0.001**	**0.049 ± 0.002**	**0.012 ± 0.001**
GNMT−	0.233 ± 0.001	0.510 ± 0.002	0.420 ± 0.010	**0.124 ± 0.002**	**0.157 ± 0.004**	0.000 ± 0.000
L-[2-^13^C]-serine	**Leu + 1 + 2 + 3**	**Gly + 1**	**Ser + 1 ^4^**	**Ser + 2 ^4^**	**dT + 1 ^4^**	**Met + 1 ^4^**
L02	0.237 ± 0.001	0.463 ± 0.011	0.591 ± 0.016	0.000 ± 0.000	**0.004 ± 0.0003**	0.000 ± 0.000
Skep1	0.228 ± 0.004	0.539 ± 0.012	0.687 ± 0.002	0.000 ± 0.000	0.000 ± 0.000	0.000 ± 0.000
HepG_2_	0.215 ± 0.001	0.398 ± 0.004	0.484 ± 0.006	0.000 ± 0.000	0.000 ± 0.000	0.000 ± 0.000
An042	0.212 ± 0.002	0.351 ± 0.009	0.365 ± 0.008	0.000 ± 0.000	**0.004 ± 0.002**	0.001 ± 0.002
GNMT+	0.255 ± 0.000	0.429 ± 0.001	0.514 ± 0.002	0.000 ± 0.000	**0.012 ± 0.001**	0.000 ± 0.000
GNMT−	0.229 ± 0.000	0.407 ± 0.028	0.505 ± 0.013	**0.018 ± 0.002**	**0.041 ± 0.001**	0.000 ± 0.000

^1^ Data are expressed as mean ± SD (*n* = 2–3). Isotopic enrichments in cellular proteins or DNA were determined, as described in the experiment procedures in Section 4. ^2^ Six cell-lines were investigated: Transformed cell-line from normal adult human hepatocyte, L02; human hepatoma cell-line, Skep1, and HepG2; human hepatoma cell-line with GNMT transfection (GNMT+); and GNMT null cell-line, GNMT−; human hepatoma cell-line with GNMT and MAT1a transfection, An042. ^3^ All cells were cultured in minimum essential medium (MEM), supplemented with B_12_ and non-essential amino acid (NEAA), as described in the experiment procedures under the “Method” section. Cells were supplemented with [2-^13^C]glycine (0.667 mM, 100% of total glycine) or L-[2-^13^C]serine (0.667 mM, 100% of total glycine), and L-[5,5,5-^2^H_3_] leucine (190 μM). ^4^ Isotopic enrichments derived from [2-^13^C]glycine and L-[2-^13^C]serine via mitochondrial glycine cleavage system are shown as the M + 2 specie of serine (Ser + 2) and in the M + 1 specie of thymidine (dT + 1) and methionine (Met + 1). The metabolic fate of [2-^13^C]-carbon is illustrated in Figure 2 and Figure 3. The data in grey indicate the enrichments of GCS derived formate using [2-^13^C]glycine tracer or L-[2-^13^C]serine tracer.

**Table 2:** Incorporation of [2-^13^C]glycine in serine, dTMP, and methionine via glycine cleavage system.

				From GCS Derived Formate ^3^			
	Gly + 1	Ser + 1	deAla + 1 ^4^	**Ser + 2** ^3^	**deAla + 2** ^3,4^	**dT + 1** ^3^	**Met + 1** ^3^
GNMT+							
MEM ^2^	0.567 ± 0.012	0.404 ± 0.011	0.379 ± 0.015	**0.044 ± 0.003**	**0.039 ± 0.001**	**0.044 ± 0.001**	0.001 ± 0.002
MEM + B12 ^2^	0.489 ± 0.010	0.377 ± 0.004	0.353 ± 0.006	**0.035 ± 0.001**	**0.033 ± 0.0002**	**0.049 ± 0.002**	**0.012 ± 0.001**
MEM + B12 + NEAA ^2^	0.452 ± 0.009	0.345 ± 0.005	0.322 ± 0.005	**0.014 ± 0.001**	**0.014 ± 0.001**	**0.012 ± 0.001**	**0.009 ± 0.001**
GNMT−							
MEM ^2^	0.579 ± 0.019	0.458 ± 0.002	0.433 ± 0.003	**0.116 ± 0.0003**	**0.107 ± 0.0001**	**0.134 ± 0.001**	0.000 ± 0.000
MEM + B12 ^2^	0.510 ± 0.002	0.420 ± 0.010	0.385 ± 0.001	**0.124 ± 0.002**	**0.109 ± 0.001**	**0.157 ± 0.004**	0.000 ± 0.000
MEM + B12 + NEAA ^2^	0.469 ± 0.004	0.390 ± 0.001	0.370 ± 0.004	**0.062 ± 0.0004**	**0.058 ± 0.001**	**0.096 ± 0.001**	0.001 ± 0.001
(**A**) Isotopic enrichments from [2-^13^C]glycine in serine, dTMP, and methionine ^1,2,3^							
				**From GCS Derived Formate** ^3^			
Nutritional effects ^5,7^	Gly + 1	Ser + 1	deAla + 1	**Ser + 2** **^3^**	**deAla + 2** **^3,4^**	**dT + 1** **^3^**	**Met + 1** **^3^**
GNMT+							
MEM + B12 vs. MEM	**−14%**	**−7%**	**−7%**	**−20%**	**−15%**	**+11%**	**+1100%**
MEM + B12 + NEAA vs. MEM	**−20%**	**−15%**	**−15%**	**−68%**	**−64%**	**−73%**	**+800%**
GNMT−							
MEM + B12 vs. MEM	**−12%**	**−8%**	**−11%**	7%	2%	**+17%**	ND
MEM + B12 + NEAA vs. MEM	**−19%**	**−15%**	**−15%**	**−47%**	**−46%**	**−28%**	ND
(**B**) Vitamin B12 and NEAA supply affect C2-glycine derived formate utilization in serine, dTMP, methionine ^5^							
				**From GCS Derived Formate** ^**3**^			
GNMT effects ^6,7 (GNMT+ vs. GNNT-)^	Gly + 1	Ser + 1	deAla + 1	**Ser + 2** **^3^**	**deAla + 2 ^3,4^**		**dT + 1** **^3^**
in MEM	−2%	**−12%**	**−12%**	**−62%**	**−64%**		**−67%**
in MEM + B12	−4%	**−10%**	**−8%**	**−72%**	**−70%**		**−69%**
in MEM + B12 + NEAA	−4%	**−12%**	**−13%**	**−77%**	**−76%**		**−88%**
(**C**) GNMT expression affect C2-glycine derived formate utilization in serine and dTMP ^6^							

^1^ Data are expressed as mean ± SD (*n* = 2–3). Isotopic enrichments in cellular proteins or DNA were determined, as described in the experiment procedures in Section 4. ^2^ GNMT overexpression cells (GNMT+), and GNMT null cells (GNMT−) were cultured in minimum essential medium (MEM), supplemented with or without B_12_ and non-essential amino acid (NEAA, 0.281 mM L-Alanine, 0.379 mM L-Asparagine, 0.226 mM L-Aspartic acid, 0.510 mM L-Glutamic Acid, 0.348 mM L-Proline), as described in the experiment procedures under the “Method” section. Cells were supplemented with [2-^13^C]glycine (0.667 mM, 100% of total glycine) or L-[2-^13^C]serine (0.667 mM, 100% of total glycine), and L-[5,5,5-^2^H_3_] leucine (190μM) for 72h before harvest. ^3^ Isotopic enrichments derived from [2-^13^C]glycine via mitochondrial glycine cleavage system are detected in the M + 2 specie of serine (Ser+2) and in the M + 1 specie of thymidine (dT + 1) and methionine (Met + 1). The metabolic fate of [2-^13^C]-carbon is illustrated in Figure 2 and Figure 3. The data in grey indicate the enrichments of GCS derived formate using [2-^13^C]glycine tracer or L-[2-^13^C]serine tracer. ^4^ Isotopic enrichments in dehydroalanine (deAla) were also determined; during derivatization of serine for GC analysis, most of the serine is converted to dehydroalanine, with loss of the proton on the C2 position. Consequently, the *m*/*z* distribution of deAla isotopes can provide the isotopic distributions at the C3 position of serine [29]. ^5^ The percent change of isotopic enrichments were calculated by comparing to that of the same cell-line cultured in MEM. ^6^ The percent change of isotopic enrichments were calculated by comparing to that of GNMT− cells in the same culture condition. ^7^ The percent change marked in bold indicates significantly increased or decreased.

## Abbreviations

Abbreviations for all figures: DHF: dihydrofolate; THF: tetrahydrofolate; CH_3_THF: 5-methyl tetrahydrofolate; 5,10-CH_2_THF: 5,10 methylene tetrahydrofolate; 5,10 CH=THF: 5,10 methenyl tetrahydrofolate;10-CHO-THF:10-formyl tetrahydrofolate; adoMet: *S*-adenosylmethionine; adoHcy: *S*-adenosylhomocysteine; dUMP: deoxyuridine monophosphate; dTMP: deoxythymidylate monophosphate; dA: deoxyadenine; dG: deoxyguanine; GAR: glycinamide ribotide; AICAR: 5-aminoimidazole-4-carboxamide ribotide; MTR: methionine synthase; MTHFR: methylene tetrahydrofolate reductase; SHMT: serine hydroxymethyltransferase; GNMT: glycine N-methyltransferase; MTR: methionine synthase; MAT: *S*-adenosylmethionine synthase; SAHH: *S*-adenosylhomocysteinehydrolase; TS: thymidylate synthase; DHFR: dihydrofolate reductase; THF: tetrahydrofolate; AICART: aminoimidazolecarboxamideribonucleotidetransformylase; GART: glycinamideribonucleotidetransformylase; Sar: sarcosine; Hcy: homocysteine; MTHFD1:methylenetetrahydrofolate dehydrogenase 1; MTHFD2: methylenetetrahydrofolate dehydrogenase 2; MTHFD2L: methylenetetrahydrofolate dehydrogenase 2–like; *GLDC*: Glycine decarboxylase; PLP: Pyridoxal phosphate; SHMT1: Serine Hydroxymethyltransferase 1; SHMT2: Serine Hydroxymethyltransferase 2; TS: Thymidine synthesis; DHFR: Dihydrofolate reductase; GCS: Glycine cleavage system.
